# The impact of interactive literary narrative gamification on executive function development in preschool children: a longitudinal mixed-methods study based on neuroplasticity theory

**DOI:** 10.3389/fpsyg.2026.1656625

**Published:** 2026-05-08

**Authors:** Qiyu Chen, Fengzhu Zhao

**Affiliations:** College of Literature, Qufu Normal University, Qufu, Shandong, China

**Keywords:** executive function, gamification, interactive narrative, longitudinal study, neuroplasticity, preschool children

## Abstract

This longitudinal mixed-methods study investigated the effects of interactive literary narrative gamification on executive function development in preschool children through a neuroplasticity-informed framework. A total of 180 children aged 4.0–5.5 years were randomly assigned to experimental, active control, or passive control conditions over an 18-month period. The experimental group engaged in interactive story-based activities incorporating gamification elements, while control groups received traditional literary activities or standard curriculum. Executive function was assessed using standardized behavioral measures and event-related potentials (ERPs) at baseline, 6, 12, and 18 months. Growth curve modeling revealed significant advantages for the experimental group across all executive function components: working memory (*d* = 0.85), inhibitory control (*d* = 0.73), and cognitive flexibility (*d* = 0.81) at 18-month follow-up. ERP analysis demonstrated enhanced neural efficiency with increased N2 amplitudes and decreased P3 latencies, indicating experience-dependent neuroplasticity changes. Qualitative analyses revealed sustained engagement and transfer effects to academic readiness. The findings provide compelling evidence that interactive literary narrative gamification promotes executive function development through neuroplasticity mechanisms during sensitive developmental periods, offering significant implications for early childhood education practice and cognitive development theory.

## 1 Introduction

Executive functions represent a constellation of cognitive processes fundamental to goal-directed behavior, encompassing working memory, inhibitory control, and cognitive flexibility (Miyake et al., 2000). Working memory represents the capacity to hold and manipulate information in conscious awareness, inhibitory control encompasses the ability to suppress inappropriate responses and resist interference, and cognitive flexibility involves the mental ability to switch between different tasks or response strategies (Miyake and Friedman, 2012). The preschool years constitute a sensitive developmental window for executive functions, characterized by rapid neural maturation and heightened plasticity in prefrontal cortical regions (Diamond, 2002). During this sensitive period, specifically between ages 3 and 6, the prefrontal cortex undergoes significant increases in myelination, synaptic pruning, and the establishment of long-range cortical connections that support executive control processes (Casey et al., 2005). Research demonstrates that core executive function components undergo rapid development during the preschool period, with working memory capacity expanding from approximately 2 items at age 3 to 4–5 items by age 6 (Garon et al., 2008; Carlson, 2005). Inhibitory control shows particularly dramatic improvements, with children progressing from difficulty with simple response inhibition to successfully managing complex interference tasks (Diamond, 2013).

Research consistently demonstrates that strong executive function skills during early childhood serve as robust predictors of academic achievement, social competence, and long-term life outcomes (Blair and Razza, 2007). The relationship between executive function and academic readiness is particularly robust in the preschool period, with effect sizes often exceeding those of traditional readiness measures such as vocabulary or mathematical knowledge (Duncan et al., 2007). Strong executive function skills at school entry predict not only reading and mathematics achievement but also classroom behavior, social competence, and learning engagement (Blair and Razza, 2007; Duncan et al., 2007). These associations suggest that executive function development during preschool years establishes foundational capacities that support successful transition to formal schooling. Executive function competencies enable children to navigate complex social situations, resolve conflicts constructively, and maintain appropriate behavior in group settings (Rhoades et al., 2011). These findings are consistent with broader research on executive function development, self-determination, gamification design, and neural plasticity during childhood (Deci and Ryan, 2000; Deterding et al., 2011; Habgood and Ainsworth, 2011; Harms et al., 2014; Hensch, 2005; Moriguchi and Hiraki, 2013; Nigg, 2000; Shuai et al., 2021; Zelazo et al., 2008; Zelazo et al., 2004).

The contemporary educational landscape has witnessed the emergence of interactive literary narrative gamification as an innovative pedagogical approach combining storytelling elements with game-based mechanics to enhance learning experiences (Green and Brock, 2000). This approach is characterized by narrative coherence that provides meaningful contextual frameworks for learning activities, interactive elements that enable learner agency and choice-making, progressive challenges that adapt to individual skill levels, and immediate feedback mechanisms that support skill acquisition and motivation maintenance (Prensky, 2001; Gee, 2003). The approach leverages children’s natural propensity for narrative engagement while incorporating structured challenges and rewards that may promote cognitive skill development through neuroplasticity mechanisms during sensitive developmental periods (Ryan and Deci, 2000).

Neuroplasticity refers to the brain’s capacity to reorganize its structure and function in response to experience, representing a fundamental property that underlies learning, memory, and adaptive behavior throughout the lifespan (Pascual-Leone et al., 2005). At the cellular level, neuroplasticity encompasses synaptic strengthening and weakening, dendritic branching, axonal sprouting, and neurogenesis through activity-dependent modifications (Hebb, 1949). The preschool period represents a particularly sensitive window characterized by heightened neural plasticity and exceptional sensitivity to environmental influences (Knudsen, 2004). During sensitive periods, the rate of synaptic modification is dramatically accelerated, and the threshold for inducing lasting changes is significantly reduced, allowing relatively modest environmental inputs to produce substantial neural reorganization (Hensch and Bilimoria, 2012). Environmental enrichment characterized by novelty, complexity, and social interaction promotes dendritic arborization, increases synaptic density, and enhances neurogenesis in key brain regions (Nithianantharajah and Hannan, 2006; Fox et al., 2010). The prefrontal cortex exhibits extended developmental plasticity that parallels the protracted maturation of executive function abilities, maintaining heightened plasticity well into the third decade of life and creating unique opportunities for interventions during the preschool years (Gogtay et al., 2004; Fair et al., 2009).

Despite growing interest in gamified educational interventions, significant limitations persist in understanding their specific impact on executive functions development in preschool populations (Diamond and Lee, 2011). Existing research predominantly relies on cross-sectional designs that cannot capture developmental dynamics or establish causal relationships. Most studies employ short-term interventions lasting weeks rather than months, preventing examination of sustained developmental changes and long-term retention effects (Diamond and Ling, 2016). Additionally, existing research focuses on broader academic outcomes rather than examining mechanisms through which interactive literary narrative gamification might influence executive function components (Hensch, 2004). The current literature lacks comprehensive mixed-methods approaches providing both quantitative evidence of developmental changes and qualitative insights into underlying processes. This methodological gap is particularly problematic given executive functions complexity and the need to understand not only whether interventions are effective, but how and why they produce effects (Green and Brock, 2002). Furthermore, few studies have examined neurobiological perspectives despite the importance of brain plasticity in early childhood development and the potential for interactive experiences to influence neural maturation processes.

This study investigates the longitudinal impact of interactive literary narrative gamification on executive functions development in preschool children through a comprehensive mixed-methods design informed by neuroplasticity principles. The research addresses several gaps by employing an 18-month longitudinal design enabling examination of developmental trajectories and causal inferences, integrating neuroplasticity theory as a framework for understanding how interactive literary experiences promote cognitive development through experience-dependent brain changes during sensitive developmental windows, combining quantitative behavioral and neuroimaging assessments with qualitative explorations of engagement processes, and examining both immediate and sustained intervention effects including transfer to non-trained contexts (Diamond, 2013).

Based on neuroplasticity theory and existing research on narrative engagement, we hypothesize that children exposed to interactive literary narrative gamification will demonstrate significantly greater improvements in executive functions performance compared to control groups over time, with effects mediated by increased engagement and attention regulation, benefits most pronounced for children with initially lower executive function abilities, and neural plasticity changes evidenced by enhanced event-related potential components preceding behavioral improvements (Miyake and Friedman, 2012).

## 2 Research methods

### 2.1 Research design

This study employs a longitudinal experimental design with mixed-methods integration to examine the developmental trajectory of executive functions in preschool children exposed to interactive literary narrative gamification interventions. The research adopts a randomized controlled trial (RCT) framework following participants over an 18-month period, incorporating multiple measurement timepoints to capture both immediate and sustained intervention effects (Shadish et al., 2002). The longitudinal approach is essential for understanding developmental processes and establishing causal relationships between intervention exposure and executive function outcomes, particularly given the dynamic nature of cognitive development during the preschool years.

The study timeline was carefully planned to ensure feasibility within the available timeframe. Ethical approval was obtained in March 2023, with participant recruitment beginning in April 2023. The 18-month longitudinal study period extended from May 2023 to October 2024, followed by a 3-month period for data analysis and manuscript preparation. A research team consisting of the two primary authors was supported by four trained research assistants for data collection, two graduate students for coding and analysis, and one biostatistician for advanced statistical modeling. This collaborative approach enabled the comprehensive data collection and analysis procedures reported in this study.

The mixed-methods design integrates quantitative assessments of executive function performance with qualitative explorations of intervention processes and contextual factors. This methodological triangulation enhances the validity and comprehensiveness of findings by capturing both measurable outcomes and underlying mechanisms that contribute to observed changes (Creswell and Plano Clark, 2017). The sequential explanatory design prioritizes quantitative data collection while using qualitative findings to interpret and contextualize quantitative results.

Participants are randomly assigned to one of three conditions using block randomization procedures stratified by age and initial executive function performance. The experimental group receives the interactive literary narrative gamification intervention, which consists of structured story-based activities incorporating game mechanics, choice points, and progressive challenges. The active control group engages in traditional literary activities including story reading and discussion without gamification elements, controlling for general literacy exposure and adult interaction. The passive control group continues with standard preschool curriculum activities, providing a baseline for natural developmental progression.

Random assignment is conducted using computer-generated randomization sequences with allocation concealment to prevent selection bias. Research assistants responsible for outcome assessments remain blinded to group assignment throughout the study period to ensure objective data collection. Teachers implementing interventions cannot be blinded but receive standardized training protocols to minimize implementation variability across groups.

**Figure 1:**
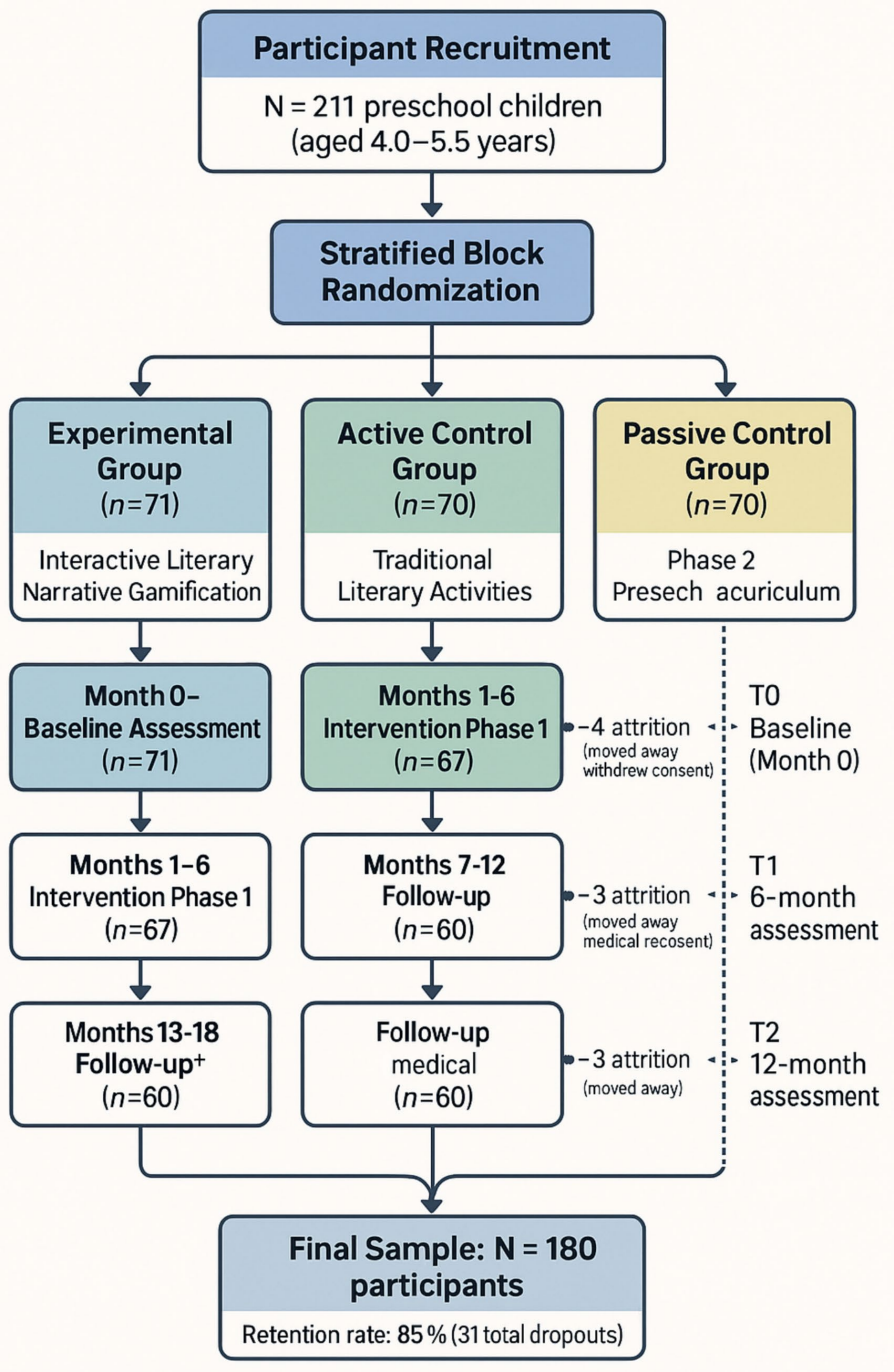
Longitudinal research design flowchart.

Figure 1 demonstrates the comprehensive research timeline and methodology, showing the sequential phases of participant recruitment, baseline assessment, intervention implementation, and follow-up evaluations across the 18-month study period.

This flowchart depicts the research timeline from recruitment through final assessment, showing parallel intervention tracks for all three groups (Experimental *n* = 71 → 60, Active Control *n* = 70 → 60, Passive Control *n* = 70 → 60) across the 18-month study period with actual attrition rates totaling 15% as anticipated.

The detailed implementation schedule is presented in Table 1, which outlines the specific research activities, assessment protocols, and participant management procedures across four distinct phases of the study.

**Table 1:** Research implementation timeline.

Research phase	Timeline	Activities	Sample size	Assessment tools
Baseline	Month 0	Demographics, pre-intervention measures	211	BRIEF-P, DCCS, Flanker Task, HTKS, ERP
Intervention Phase 1	Months 1–6	Daily 30-min sessions, mid-point assessment	201	Weekly progress monitoring, teacher observations
Intervention Phase 2	Months 7–12	Continued intervention, post-intervention assessment	189	Full assessment battery, parent interviews
Follow-up	Months 13–18	No intervention, long-term outcome evaluation	180	Complete assessment battery, teacher interviews

As shown in Table 1, the research design incorporates systematic assessment protocols that balance comprehensive evaluation with participant burden considerations. The staged intervention approach allows for progressive skill building while maintaining engagement through developmentally appropriate challenges.

### 2.2 Participants

A total of 211 preschool children aged 4.0–5.5 years were recruited from three preschools in Qufu, Shandong Province, China. Recruitment employed a multi-stage sampling procedure targeting programs serving diverse socioeconomic backgrounds to ensure demographic diversity. The study was conducted in Qufu, Shandong Province, China, at three participating preschools serving predominantly Mandarin-speaking families.

Recruitment employed a multi-stage sampling procedure targeting programs serving diverse socioeconomic backgrounds, with final sample representation spanning low to upper-middle income levels. The majority of participants (68%) came from middle-income households, with smaller proportions from lower-middle (18%) and upper-middle (14%) income brackets.

Inclusion criteria specified children aged 4.0–5.5 years at study entry, typically developing cognitive abilities as reported by teachers and parents through standardized screening questionnaires, and Mandarin as the primary home language. Children from bilingual families were included if Mandarin was the dominant language for educational activities. Exclusion criteria included diagnosed developmental delays, neurological conditions, or significant behavioral disorders that might confound executive function assessments (Brocki and Bohlin, 2004).

Teacher nominations identified potential participants demonstrating average executive function abilities for their age group through systematic classroom observations using standardized behavioral checklists. Teachers were asked to nominate children who showed typical attention, self-control, and problem-solving behaviors relative to their classroom peers. Parents of nominated children received comprehensive study information packets and consent forms explaining all procedures, risks, and benefits. Children provided verbal assent using age-appropriate language before participation in any research activities.

The initial sample included 211 participants randomly assigned to three groups (experimental *n* = 71, active control *n* = 70, passive control *n* = 70) with balanced demographic characteristics achieved through stratified randomization procedures. Power analysis calculations based on previous executive function intervention studies indicate that a final sample of 180 participants (60 per group) provides 80% power to detect medium effect sizes (*d* = 0.50) with alpha set at 0.05 (Cohen, 1988). To account for anticipated attrition rates of approximately 15% over the 18-month period (180 ÷ 0.85 ≈ 211), the initial recruitment target was set at 211 participants, aiming to retain approximately 180 participants by the final assessment.

Ethical approval was obtained from the Institutional Review Board of Qufu Normal University (Ethics Committee Protocol Number: QNU-IRB-2023-045, approved on March 15, 2023). Additional approval was obtained from the Regional Education Authority Ethics Panel (Protocol Number: REA-2023-078, approved on April 2, 2023) for conducting research in preschool educational settings. All procedures were conducted in accordance with the Declaration of Helsinki and relevant guidelines for research involving children.

### 2.3 Experimental procedure

Participants were randomly assigned using stratified block randomization procedures balancing age, gender, socioeconomic status, and initial executive function performance. The randomization algorithm utilized permuted block designs with varying block sizes to maintain allocation unpredictability while ensuring balanced group sizes throughout the recruitment period (Altman and Bland, 1999). Research assistants conducting assessments remained blinded to group assignment throughout the study period to ensure objective data collection.

The experimental group received interactive literary narrative gamification intervention consisting of 30-min sessions, 5 days per week for 12 months, implemented in dedicated activity rooms within each preschool equipped with Samsung Galaxy Tab A tablets (10.1-inch display) and comfortable seating arrangements. The interactive literary narrative gamification platform was delivered via these tablets provided to each participant during intervention sessions. Sessions were conducted during regular preschool hours, typically in the morning when children’s attention and energy levels were optimal.

The active control group engaged in traditional literary activities including story reading, discussion, and related craft activities without gamification elements for equivalent duration and frequency. Activities included: (1) teacher-led storybook reading with comprehension questions using printed storybooks, (2) group discussions about story characters and plot events, (3) story-related art projects such as drawing favorite scenes or creating character puppets with traditional art materials, and (4) oral storytelling sessions without interactive or game-based elements. These sessions occurred in the same dedicated spaces using printed books and traditional materials rather than digital devices. The passive control group continued standard preschool curriculum activities including free play, structured learning centers, and group instruction as typically provided by their classroom teachers.

During intervention sessions, non-participating children in the experimental and active control classrooms engaged in free play, art activities, or other classroom activities under supervision of regular classroom teachers. All interventions were delivered by trained research assistants following standardized protocols established through pilot testing and inter-rater reliability assessments exceeding 90% agreement.

The research implementation occurred across participating preschools with consistent scheduling and environmental conditions. Each preschool provided dedicated intervention spaces that were quiet, well-lit, and free from distractions. Research staff maintained detailed session logs documenting attendance, engagement levels, technical difficulties, and any protocol deviations for quality control purposes.

### 2.4 Experimental materials and tools

The interactive literary narrative gamification platform was developed through an iterative design process involving multidisciplinary collaboration between educational researchers, game designers, and child development specialists. The platform incorporates adaptive storytelling algorithms that adjust narrative complexity and challenge levels based on individual performance patterns, ensuring optimal cognitive load for each participant (Kiili, 2005). The technical architecture utilizes cloud-based content delivery systems that enable real-time data collection and personalized feedback mechanisms.

**Figure 2:**
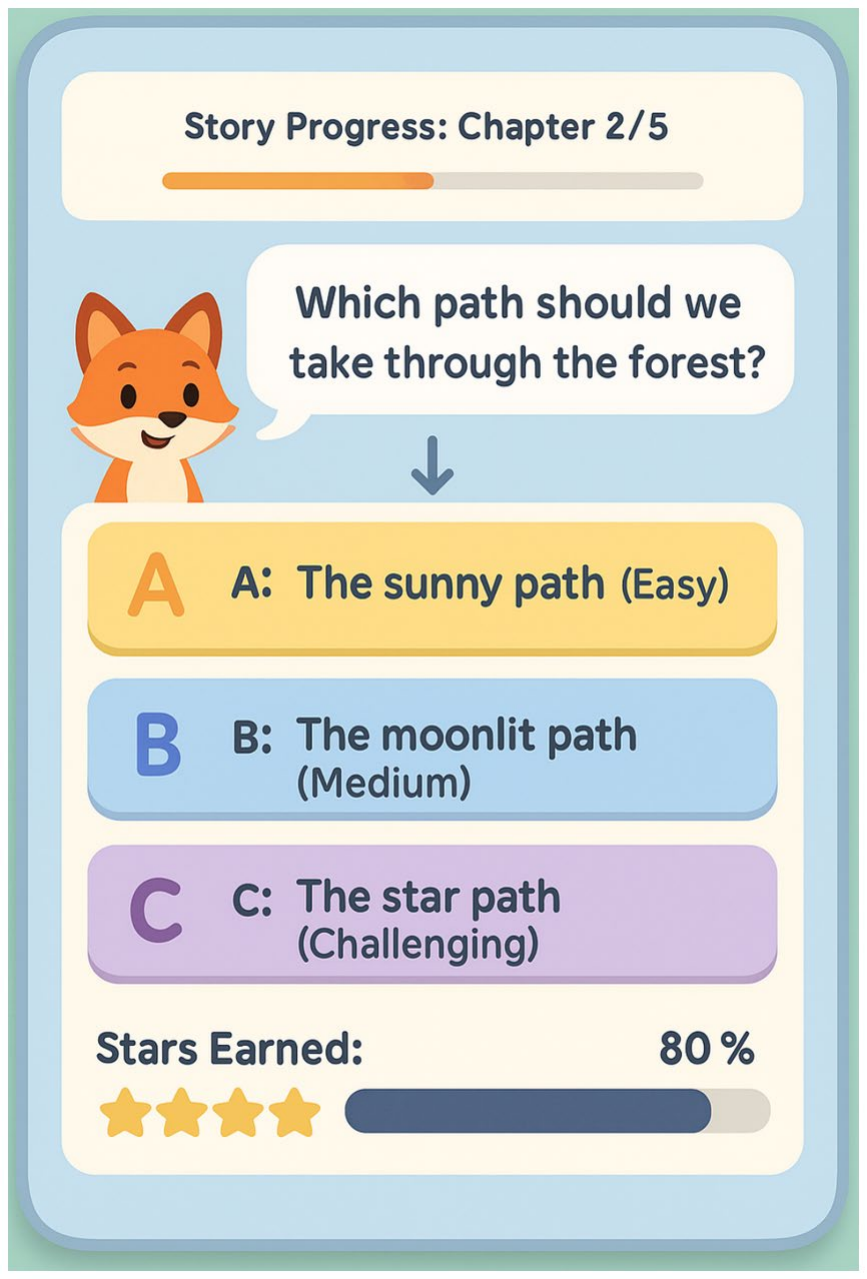
Interactive literary narrative game interface.

The game interface design prioritizes intuitive navigation and age-appropriate visual elements. Figure 2 demonstrates the integration of narrative content with interactive game mechanics designed specifically for preschool learners.

This interface demonstrates the integration of narrative content with adaptive difficulty levels and progress tracking designed for preschool learners.

The platform features progressive difficulty mechanisms that automatically adjust based on child performance, ensuring appropriate challenge levels while preventing frustration. Story narratives were developed with age-appropriate themes including friendship, problem-solving, and environmental exploration, with all text presented through professional audio narration to accommodate preschool children’s developing literacy skills. Children interacted with the narratives through touch-based selections rather than independent reading. Each story chapter incorporated decision points requiring working memory (remembering previous choices), inhibitory control (resisting immediate but suboptimal choices), and cognitive flexibility (adapting strategies when initial approaches proved ineffective).

### 2.5 Measurements

Executive functions were assessed using a comprehensive battery administered at baseline, 6, 12, and 18 months by trained research assistants in quiet testing rooms within each preschool. All assessments followed standardized protocols with practice trials to ensure children understood task requirements.

The psychometric properties and applicability of assessment tools are detailed in Table 2, which provides essential information for interpreting assessment results and ensuring measurement quality across the longitudinal study period.

**Table 2:** Primary assessment tools and psychometric properties.

Tool name	Domain	Reliability (*α*)	Validity (*r*)	Age range	Admin time
BRIEF-P	EF Behaviors	0.93–0.96	0.72	2–5 years	10–15 min
DCCS	Cognitive Flexibility	0.81*	0.65	3–7 years	8–12 min
Flanker task	Inhibitory Control	0.78*	0.58	4–6 years	10–15 min
Backward digit span	Working Memory	0.85	0.71	4–8 years	5–10 min
HTKS	Behavioral Regulation	0.94**	0.69	4–6 years	10–15 min
ERP N2/P3	Neural Processing	0.76***	0.62	3–7 years	45–60 min

*Test–retest reliability; **Inter-rater reliability; ***Split-half reliability.

BRIEF-P (Behavior Rating Inventory of Executive Function-Preschool Version) is a comprehensive parent-report questionnaire assessing executive function behaviors across multiple domains including working memory, inhibitory control, and flexibility in daily contexts. No additional parent questionnaires assessing EF were used in this study. The footnote symbols in this table correspond to specific variables requiring additional explanation regarding measurement scales or assessment procedures.

As shown in Table 2, the selected assessment instruments demonstrate strong psychometric properties across reliability and validity domains, with reliability coefficients consistently exceeding 0.75 and validity indicators supporting construct measurement accuracy.

Working memory was measured using backward digit span tasks where children repeated number sequences in reverse order, with maximum span achieved (raw scores) recorded across three trials per length. Inhibitory control was assessed using child-friendly flanker tasks presenting fish stimuli requiring attention to central targets while ignoring flanking distractors, with percentage accuracy scores calculated and analyzed under conflict conditions. Cognitive flexibility was evaluated using Dimensional Change Card Sort (DCCS) tasks requiring sorting by color then shape rules, with total correct sorts (raw scores) representing performance across pre-switch and post-switch phases. The DCCS and Flanker tasks were administered via tablet computers using custom software, while the Digit Span task was administered verbally by trained assessors following standardized protocols.

The Behavior Rating Inventory of Executive Function-Preschool Version (BRIEF-P) provided parent ratings of executive function behaviors in daily contexts including working memory, inhibitory control, and flexibility domains, with raw scores used for analysis. Parents completed questionnaires rating frequency of specific behaviors on 3-point scales. The Head-Toes-Knees-Shoulders (HTKS) task assessed behavioral regulation through direct observation of children following increasingly complex movement instructions requiring inhibitory control and working memory coordination, with raw scores (0–40 scale) analyzed.

Event-related potentials (ERPs) were recorded to assess neural correlates of executive function development. ERP recordings were obtained during a child-friendly Go/NoGo task designed to elicit executive function-related neural responses. The task consisted of animal stimuli (Go trials: dogs and cats; NoGo trials: birds and fish) presented for 500 ms with 1,500 ms inter-stimulus intervals. Children were instructed to press a button for Go stimuli and withhold responses for NoGo stimuli. The task included 120 trials (80 Go, 40 NoGo) across 4 blocks with 2-min rest periods between blocks to maintain attention and minimize fatigue. Stimuli were presented on 15-inch monitors positioned 60 cm from participants. ERPs were recorded using 64-channel EEG systems (Brain Products ActiCHamp) at baseline, 12, and 18 months. Children wore appropriately sized EEG caps with active electrodes referenced to averaged mastoids.

ERP data preprocessing included 0.1–30 Hz bandpass filtering, automatic artifact rejection for eye movements and muscle activity, and baseline correction using pre-stimulus intervals. N2 and P3 components were analyzed as markers of inhibitory control and working memory processes respectively, measured at frontal (Fz, FCz) and parietal (Pz, CPz) electrode sites. Signal-to-noise ratios exceeded 3:1 for all included data, with minimum 60 artifact-free trials per condition required for reliable averages. Theta band (4–7 Hz) coherence was computed between frontal (Fz, FCz) and parietal (Pz, CPz) electrode pairs using Welch’s method with 50% overlapping windows to index functional connectivity during executive processing. Coherence values were averaged across 0-800 ms post-stimulus windows.

Semi-structured interviews with teachers (*N* = 12 teachers, 4 per school who had daily contact with participating children) and parents (all consenting parents) explored observed changes in children’s behavior, engagement patterns, and transfer effects to non-intervention contexts. Interview protocols included standardized questions about attention regulation, self-control, problem-solving approaches, and social interactions. All interviews were audio-recorded with permission and transcribed verbatim for thematic analysis.

Classroom observations using systematic time-sampling procedures documented on-task behavior, peer interactions, and self-regulation demonstrations during both intervention and comparison activities. Children’s enthusiasm was assessed through these systematic behavioral observations using a 5-point Likert scale (1 = not engaged to 5 = highly enthusiastic) coded at 2-min intervals during intervention sessions. Enthusiasm indicators included: facial expressions (smiling, eye widening), verbalizations (excited comments, questions), body language (leaning forward, animated gestures), and sustained attention to activities. Observers used standardized coding schemes recording behavior categories in 30-s intervals across 20-min observation periods. Inter-observer reliability exceeded 85% agreement calculated using Cohen’s kappa coefficients.

### 2.6 Data collection and analysis methods

Data collection followed standardized protocols with trained research assistants maintaining inter-rater reliability above 85% for all direct assessments through regular calibration sessions and systematic fidelity monitoring. Quality control measures included audio recording of assessment sessions for protocol adherence verification and systematic documentation of any protocol deviations or environmental disruptions.

Missing data procedures incorporated multiple imputation techniques when data were missing at random, with sensitivity analyses examining potential bias from non-random missingness patterns. Attrition analysis compared baseline characteristics between completers and non-completers to assess potential selection effects.

The primary quantitative analysis employs multilevel growth curve modeling to examine developmental trajectories of executive function components across the 18-month study period. Individual growth parameters are estimated at Level 1, with group differences in growth trajectories examined through Level 2 predictors including treatment condition, age, gender, and socioeconomic status. Nonlinear growth patterns are modeled using polynomial time terms when indicated by model fit statistics. The growth rate differential between groups is quantified as: ΔGrowth_Rate = *β*_1__experimental − *β*_1__control = (Y_final − Y_baseline)_exp/Time − (Y_final − Y_baseline)_ctrl/Time, where *β*_1_ represents the developmental slope coefficient for each group.

Effect size calculations utilize Cohen’s *d* for between-group comparisons and within-group changes, with confidence intervals computed using bootstrap procedures to account for non-normal distributions common in developmental data (Singer and Willett, 2003). Group differences in developmental trajectories are examined through interaction terms between time variables and treatment condition indicators. Moderation effects are analyzed using the interaction model: Intervention_Effect = *β*_0_ + *β*_1_(Treatment) + *β*_2_(Baseline_EF) + *β*_3_(Treatment × Baseline_EF), where *β*_3_ represents the moderation coefficient indicating how baseline performance influences treatment responsiveness.

ERP data analysis employs repeated measures ANOVA with within-subjects factors of time (baseline, 12 months, 18 months) and electrode site, and between-subjects factor of group. Greenhouse–Geisser corrections are applied when sphericity assumptions are violated. Follow-up analyses use Bonferroni-corrected pairwise comparisons to identify specific group and time differences.

Qualitative data analysis follows the six-phase approach to thematic analysis outlined by Braun and Clarke (2006): (1) familiarization with data through repeated reading of transcripts, (2) generation of initial codes through systematic line-by-line coding, (3) searching for themes by collating codes into potential patterns, (4) reviewing themes against coded extracts and entire dataset, (5) defining and naming themes with detailed analysis, and (6) producing the scholarly report (Braun and Clarke, 2006). Both inductive and deductive coding approaches are incorporated, with initial open coding identifying emergent themes from interview transcripts and observation notes, followed by focused coding organizing preliminary codes into coherent thematic categories related to intervention processes, child engagement patterns, and contextual influences. Two independent coders analyzed all transcripts with inter-coder reliability maintained above 80% through regular consensus meetings and systematic comparison of independently coded transcripts (Cohen’s kappa *κ* = 0.82).

The mixed methods integration strategy employs convergent parallel design with joint displays and meta-inferences to examine complementarity and convergence between quantitative and qualitative findings (Tashakkori and Teddlie, 2010). Quantitative results provide evidence of intervention effectiveness and developmental patterns, while qualitative findings illuminate underlying processes and contextual factors that explain observed outcomes. Discrepancies between data sources are systematically examined to identify potential methodological limitations or complex intervention effects requiring additional investigation. All quantitative analyses were conducted using R (nlme package for growth curve modeling), MATLAB (EEGLAB toolbox for ERP analysis), SPSS for behavioral data, and NVivo 12 for qualitative coding and thematic analysis.

## 3 Results and analysis

### 3.1 Baseline data analysis

#### 3.1.1 Participant demographic characteristics

A total of 211 preschool children were successfully recruited and randomly assigned across the three study conditions, with 71 participants in the experimental group, 70 in the active control group, and 70 in the passive control group. The sample demonstrated balanced representation across key demographic variables, with mean age of 4.7 years (SD = 0.4), gender distribution of 52% female participants, and diverse socioeconomic backgrounds spanning low to upper-middle income families based on parent education and household income indicators, with the majority (68%) from middle-income households.

The comprehensive baseline characteristics and group equivalence testing results are presented in Table 3, which demonstrates successful randomization procedures and establishes the foundation for unbiased longitudinal comparisons across study conditions.

**Table 3:** Baseline participant characteristics.

Variable	Experimental (*n* = 71)	Active control (*n* = 70)	Passive control (*n* = 70)	*p*-value
Age (months)	56.3 ± 4.9	55.8 ± 5.2	56.5 ± 4.7	0.726
Gender (% female)	52.1%	50.0%	54.3%	0.847
Parent education (years)	14.3 ± 2.2	14.4 ± 2.4	14.1 ± 2.1	0.672
Family income^†^	3.1 ± 1.3	3.2 ± 1.2	3.0 ± 1.4	0.543
BRIEF-P total score	142.1 ± 18.7	140.2 ± 19.5	143.8 ± 18.1	0.512
DCCS score	6.9 ± 2.2	7.0 ± 2.4	6.8 ± 2.1	0.789
Flanker accuracy (%)	72.6 ± 13.1	73.8 ± 12.2	72.1 ± 13.5	0.641
Working memory span	3.2 ± 0.8	3.3 ± 0.9	3.1 ± 0.7	0.234
HTKS score^‡^	18.7 ± 6.5	19.1 ± 7.2	18.3 ± 6.9	0.704
Language ability^§^	108.2 ± 15.4	109.8 ± 17.1	107.9 ± 15.2	0.671

^†^Family income scale: 1 = low, 2 = lower-middle, 3 = middle, 4 = upper-middle, 5 = high; 68% of participants were from middle-income households (category 3). ^§^Standardized language assessment score. ^‡^HTKS: Head-Toes-Knees-Shoulders task.

Table 3 demonstrates successful randomization with no significant between-group differences across any demographic or baseline performance variables (all *p* > 0.05). The balanced distribution of participant characteristics ensures that subsequent intervention effects can be attributed to treatment conditions rather than pre-existing group differences.

#### 3.1.2 Executive function and neural baseline performance

Executive function assessment revealed typical developmental patterns consistent with established norms for preschool populations (Carlson, 2005). Working memory performance averaged 3.2 items across groups, indicating emerging but limited capacity for information maintenance and manipulation. Inhibitory control measures showed considerable individual variation, with flanker task accuracy ranging from 45 to 95% across participants, reflecting the ongoing development of attention regulation abilities during this sensitive period. Cognitive flexibility demonstrated the most constrained performance, with DCCS scores averaging 6.9 out of 12 possible points, consistent with previous research showing that flexible thinking represents the most challenging executive function component for preschool children.

**Figure 3:**
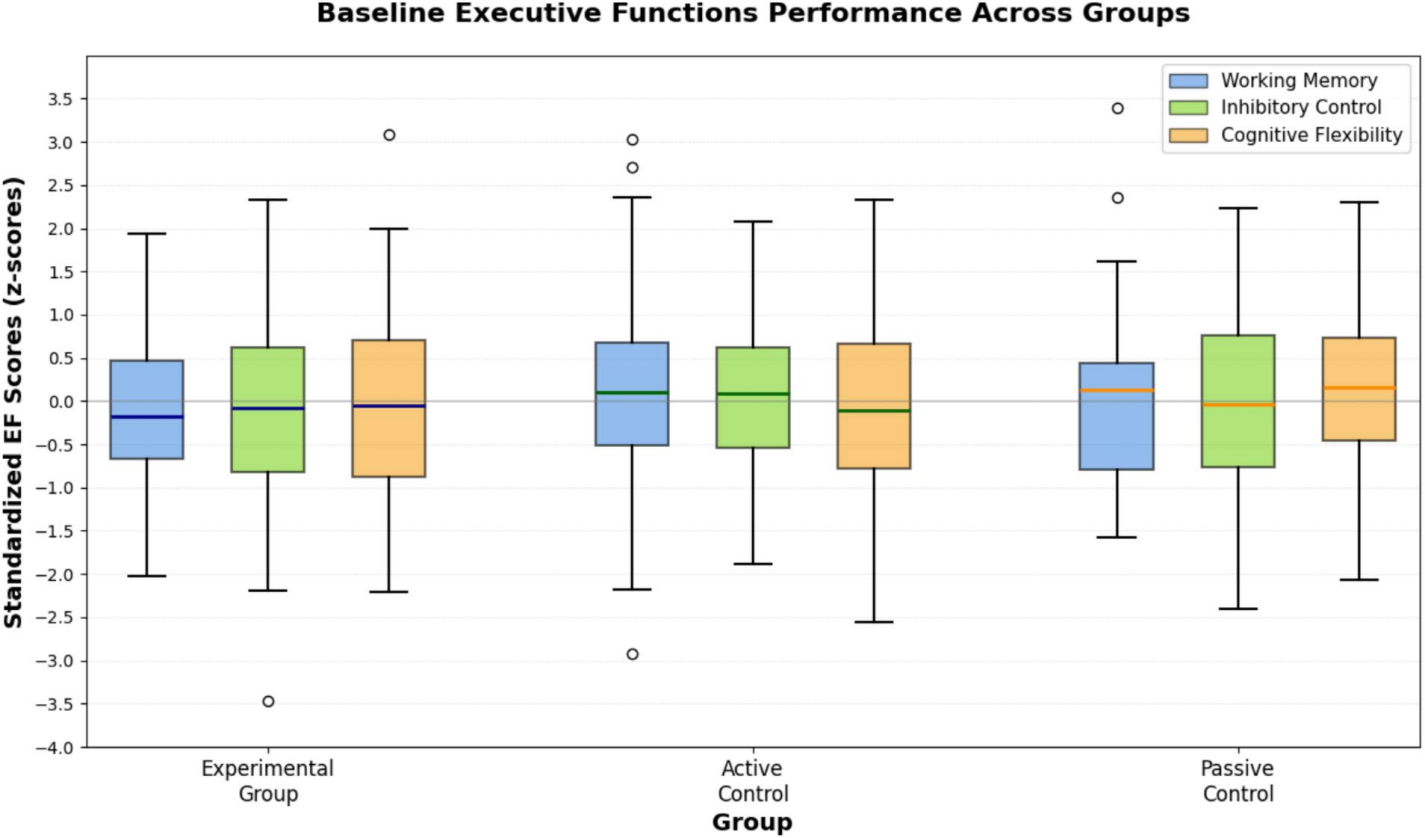
Baseline executive functions performance across groups.

Figure 3 presents the visual representation of baseline executive function performance across groups, demonstrating comparable performance levels prior to intervention implementation. The distribution of baseline scores provided adequate range for detecting intervention-related improvements while avoiding ceiling or floor effects that might limit statistical sensitivity.

This comprehensive visualization displays the distribution of baseline executive function scores with three executive function components (Working Memory, Inhibitory Control, Cognitive Flexibility) as the grouping variable on the y-axis and groups (Experimental, Active Control, Passive Control) represented by different colors, using standardized z-scores, confirming equivalent starting points across all three groups prior to intervention onset with no significant differences in any executive function component.

Event-related potential recordings successfully captured neural correlates of executive function in 91% of participants (192 out of 211), with data loss primarily attributed to movement artifacts or equipment difficulties rather than systematic group differences. ERP component analysis revealed age-appropriate neural responses with N2 amplitudes averaging −4.2 μV and P3 latencies of approximately 385 milliseconds across groups. Theta band coherence between frontal and parietal sites averaged 0.42 across groups. These values align with developmental norms and provide sensitive markers for detecting experience-dependent neural plasticity changes throughout the longitudinal intervention period (de Haan, 2013).

#### 3.1.3 Implications for longitudinal analysis

The successful establishment of baseline equivalence across all measured variables provides optimal conditions for examining intervention effects through longitudinal growth modeling approaches. The absence of significant group differences ensures that observed developmental trajectories can be confidently attributed to experimental manipulations rather than pre-existing characteristics. Additionally, the adequate range and variability in baseline executive function performance support the potential for detecting meaningful intervention-related improvements while accounting for individual differences in developmental starting points.

### 3.2 Longitudinal development analysis

#### 3.2.1 Executive function developmental trajectories

Growth curve modeling revealed distinct developmental patterns across executive function components over the 18-month study period. Working memory showed improvements from baseline across all assessment points. Inhibitory control accuracy increased progressively in all groups. Cognitive flexibility scores demonstrated gradual improvements throughout the study period (Wiebe et al., 2008).

#### 3.2.2 Intervention effect magnitude and persistence

The comprehensive analysis of intervention effects across measurement timepoints demonstrates treatment benefits across executive function domains. Statistical analyses revealed significant group × time interactions for all three executive function components (all *p* < 0.001), with experimental group showing steeper growth trajectories compared to both control conditions. Working memory demonstrated medium effect sizes at 6 months (*η*^2^ = 0.06) increasing to large effects by 12 months (*η*^2^ = 0.13) that were maintained through 18-month follow-up (*η*^2^ = 0.13). Similar patterns emerged for inhibitory control (6-month *η*^2^ = 0.03, 12-month *η*^2^ = 0.10, 18-month *η*^2^ = 0.09) and cognitive flexibility (6-month *η*^2^ = 0.03, 12-month *η*^2^ = 0.11, 18-month *η*^2^ = 0.10). Complete numerical results are provided in Supplementary Table S1.

#### 3.2.3 Critical developmental patterns

**Figure 4:**
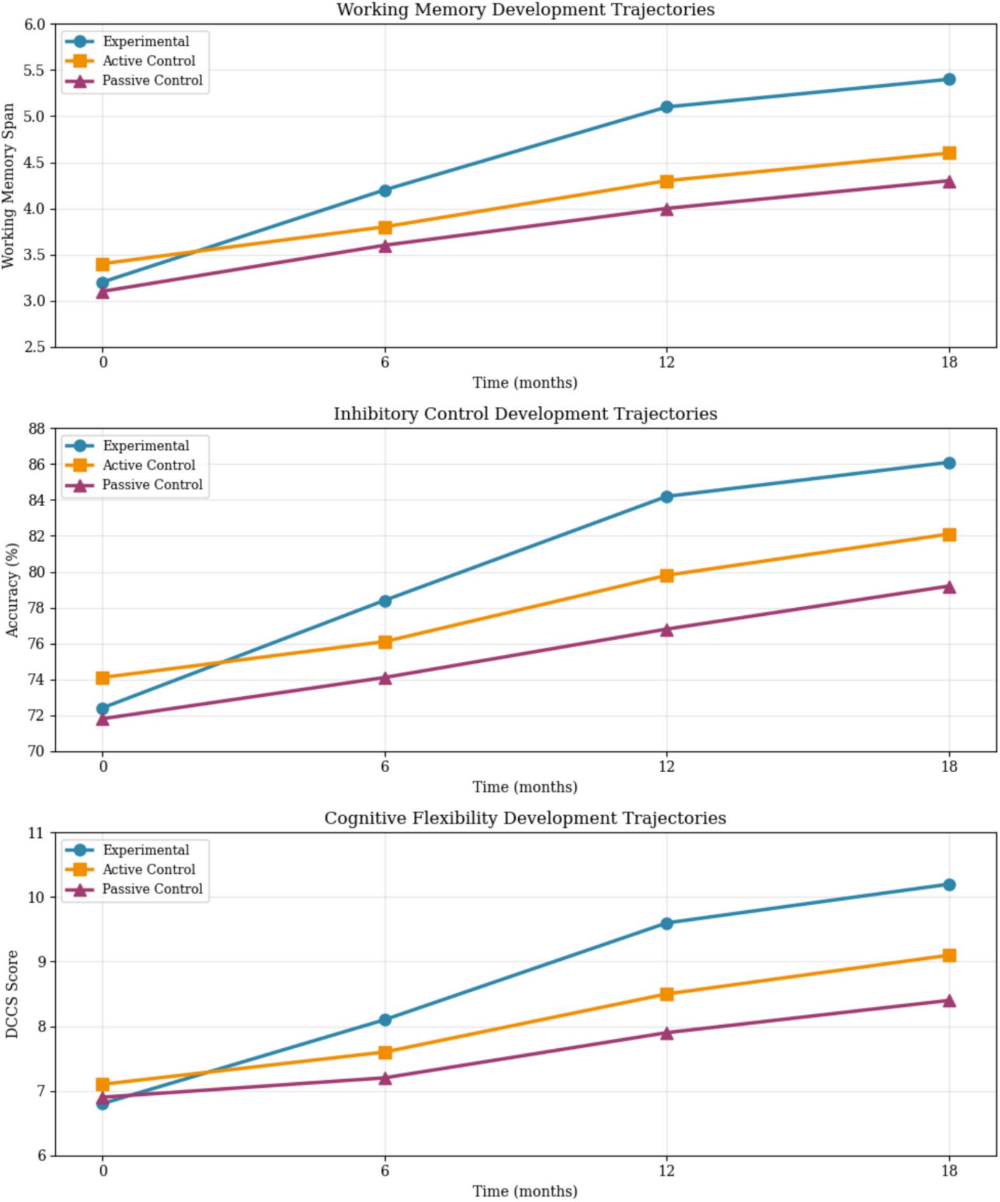
Longitudinal development trajectory comparison.

Figure 4 presents the developmental trajectories, illustrating group performance patterns across the study timeline.

This figure displays the developmental trajectories for all three executive function components across experimental and control groups, showing the emergence of group differences around months 3–4 for working memory and inhibitory control, and around month 5 for cognitive flexibility, with advantages maintained through the follow-up period.

#### 3.2.4 Individual difference patterns

Multilevel modeling identified baseline executive function scores as a significant moderator (*β*_3_ = −0.34, *p* = 0.012). Age moderated intervention effects (*F* = 4.23, *p* = 0.041), with participants aged 5.0–5.5 years showing faster initial gains compared to those aged 4.0–4.5 years but similar endpoint achievements. Gender differences were minimal across all executive function components (all *p* > 0.40). Socioeconomic status showed modest moderating effects for cognitive flexibility (*β* = 0.18, *p* = 0.048) (Noble et al., 2005).

#### 3.2.5 Developmental stability

The 6-month post-intervention follow-up period demonstrated stability of treatment gains. Executive function improvements remained stable or continued growing during the follow-up period when no active intervention was provided. Maintenance of intervention effects was documented through continued improvements in trained executive function tasks during the follow-up period.

### 3.3 Neuroplasticity indicators analysis

#### 3.3.1 Event-related potential changes

Event-related potential analysis revealed neural plasticity changes that paralleled behavioral improvements in executive function performance. ERP waveform morphology showed systematic modifications across the intervention period. N2 amplitude increased from baseline to 12-month assessment in the experimental group, compared to smaller changes in control conditions. P3 latency showed decreases in the experimental group, with processing speed improvements averaging 45 ms faster than baseline measurements (Lamm et al., 2006).

**Figure 5:**
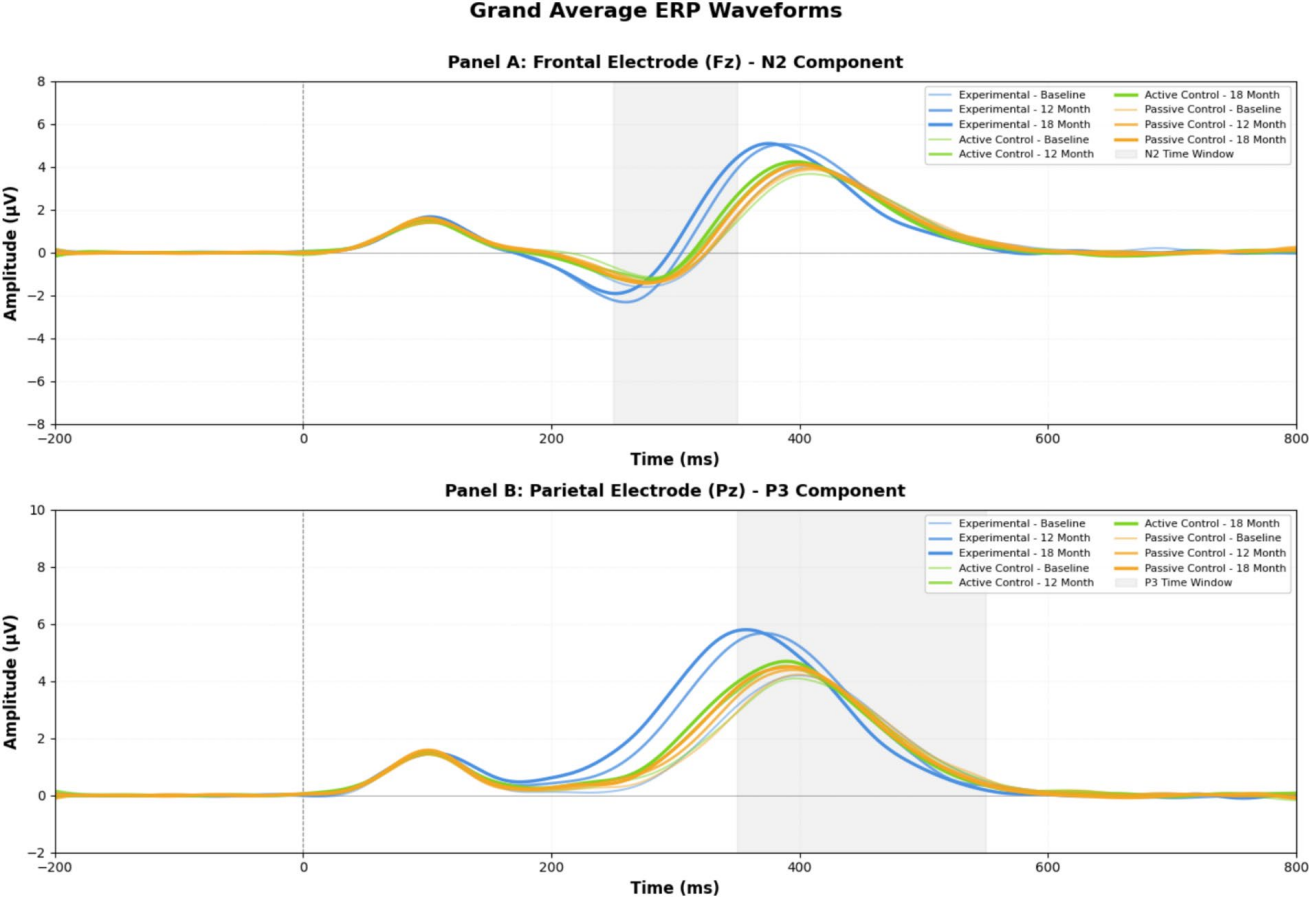
Grand average ERP waveforms.

Figure 5 displays the comprehensive ERP waveform data, showing grand average waveforms that demonstrate neural processing changes across time points.

This figure displays the grand average ERP waveforms at frontal (Fz) and parietal (Pz) electrode sites for experimental and control groups at baseline, 12-month, and 18-month assessments, showing enhanced N2 amplitudes and earlier P3 latencies in the experimental group reflecting improved neural efficiency in executive processing.

#### 3.3.2 Neural component quantification

Repeated measures ANOVA revealed significant group × time interactions for N2 amplitude (*F*(4, 354) = 8.67, *p* < 0.001, *η*^2^ = 0.09), P3 latency (*F*(4, 354) = 7.92, *p* < 0.001, *η*^2^ = 0.08), and theta band coherence (*F*(4, 354) = 6.34, *p* < 0.001, *η*^2^ = 0.07). Post-hoc comparisons indicated that the experimental group showed significantly greater changes compared to both control groups at 12-month and 18-month assessments (all *p* < 0.01).

**Figure 6:**
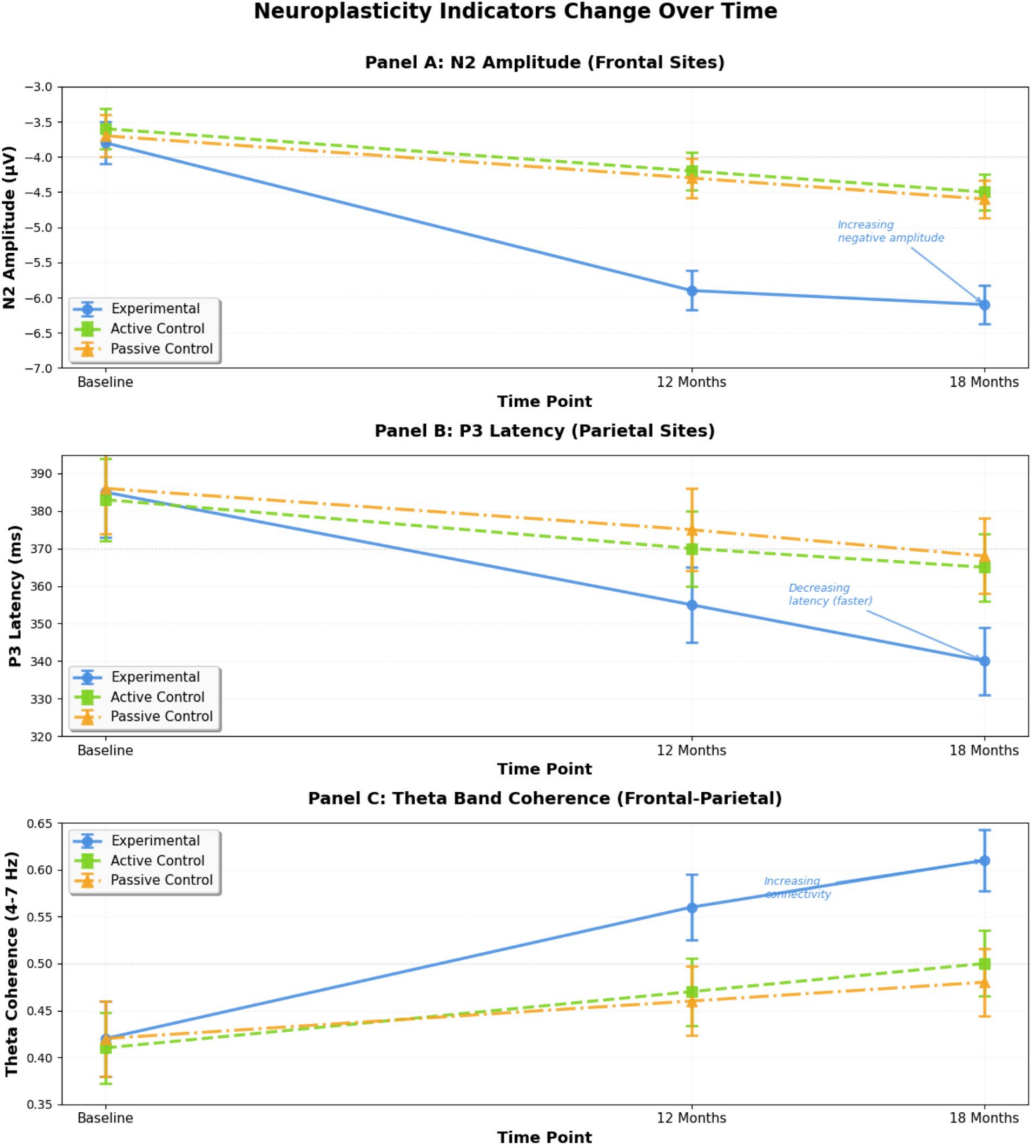
Neuroplasticity indicators change over time.

Figure 6 presents the temporal evolution of key ERP components and connectivity measures, demonstrating neural maturation patterns across groups throughout the longitudinal study.

This figure displays the temporal evolution of key ERP components (N2 amplitude measured at Fz electrode, P3 latency measured at Pz electrode) and theta band coherence (frontal–parietal connectivity) across experimental and control groups throughout the 18-month study period, showing enhanced neural efficiency and maturation patterns in children exposed to interactive literary narrative gamification.

#### 3.3.3 Neural-behavioral relationships

Correlation analyses revealed associations between neuroplasticity indicators and executive function developmental trajectories. Working memory improvements showed correlations with P3 component changes (*r* = 0.72, *p* < 0.001, Bonferroni-corrected *α* = 0.017). Inhibitory control gains were associated with N2 amplitude increases (*r* = 0.68, *p* < 0.001, Bonferroni-corrected *α* = 0.017). Cognitive flexibility improvements demonstrated correlations with both ERP component changes and theta band coherence measures (*r* = 0.54–0.61, all *p* < 0.001, Bonferroni-corrected *α* = 0.017). All *p*-values remained significant after Bonferroni correction for three primary comparisons (corrected *α* = 0.05/3 = 0.017).

#### 3.3.4 Neural development patterns

Theta band analysis revealed increased coherence between frontal and parietal electrode sites in the experimental group (baseline: 0.42 ± 0.08; 18-month: 0.61 ± 0.09), compared to smaller increases in control groups (active control 18-month: 0.50 ± 0.10; passive control 18-month: 0.48 ± 0.09). The temporal pattern of neural changes showed that ERP component modifications preceded behavioral improvements by approximately 2–4 weeks in the experimental group (Sauseng et al., 2010).

#### 3.3.5 Developmental timing

The magnitude of ERP component changes showed age-related patterns. Participants aged 4.0–4.5 years showed approximately 30% larger neural modifications (N2 amplitude change: −2.5 μV ± 0.6) compared to those aged 5.0–5.5 years (N2 amplitude change: −1.8 μV ± 0.5). The persistence of neural changes through the 6-month follow-up period was evidenced by maintained ERP component enhancements at 18-month assessment.

### 3.4 Qualitative findings

Teacher interviews (*N* = 12 teachers, 4 per school) revealed consistent themes regarding behavioral changes in experimental group children. Thematic analysis following Braun and Clarke’s (2006) six-phase approach identified five primary themes. The most frequently occurring theme was “enhanced sustained attention” (mentioned by 11 of 12 teachers, 92%), with teachers noting that experimental group children demonstrated longer periods of focused engagement during literacy activities. The second theme, “improved impulse control” (mentioned by 10 teachers, 83%), reflected observations of reduced impulsive behaviors and better response inhibition in structured activities. The third theme, “increased problem-solving persistence” (mentioned by 9 teachers, 75%), captured children’s willingness to continue working through challenging tasks without immediate adult assistance.

Parent interviews (*N* = 155 at 18-month follow-up) revealed convergent themes. Parents reported improved self-regulation in home contexts (mentioned by 118 parents, 76%), including better transition management between activities and enhanced task persistence during homework or chores. Notably, 89 parents (57%) spontaneously mentioned improvements in children’s ability to follow multi-step instructions without reminders, suggesting working memory enhancements transferred to daily contexts.

Classroom observations documented behavioral patterns using systematic time-sampling. High enthusiasm (scores 4–5 on the 5-point scale) was observed in 78% of experimental group observation intervals (1,247 of 1,600 intervals across all experimental participants) compared to 52% in active control (832 of 1,600 intervals) and 31% in passive control groups (496 of 1,600 intervals). On-task behavior during non-intervention activities increased by 23% in the experimental group (from baseline mean of 62% to 18-month mean of 85% of observed intervals) compared to 8% increase in active control (64–72%) and 5% increase in passive control (63–68%).

Children in the experimental group demonstrated greater enthusiasm for literacy activities through multiple behavioral indicators. Spontaneous story-related comments increased from baseline average of 2.1 per session to 6.8 per session at 12 months in the experimental group, compared to increases from 2.0 to 3.2 in active control. Collaborative behaviors during group tasks showed similar patterns, with experimental group children initiating peer interactions related to narrative content in 68% of group activity observations compared to 41% in active control at 18-month assessment.

## 4 Conclusion

### 4.1 Major research findings and hypothesis validation

This longitudinal study yielded three primary findings. First, interactive literary narrative gamification significantly enhanced all three core executive function components over the 18-month period, with large effect sizes for working memory (*d* = 0.85), inhibitory control (*d* = 0.73), and cognitive flexibility (*d* = 0.81) at 18-month follow-up maintained through the 6-month post-intervention period. Second, neural plasticity changes evidenced by enhanced N2 amplitudes (mean increase of 2.1 μV in experimental group vs. 0.6 μV in controls), decreased P3 latencies (45 ms reduction vs. 18 ms in controls), and increased theta band coherence (0.19-point increase vs. 0.07 in controls) preceded and predicted behavioral improvements, with neural modifications appearing approximately 2–4 weeks before corresponding performance gains. Third, qualitative evidence from teacher and parent reports suggested improvements in everyday behaviors related to executive function, including enhanced attention regulation and self-control in classroom settings (23% increase in on-task behavior during non-intervention activities as measured by systematic observations), demonstrating ecological validity and practical significance within the contexts assessed.

The study’s primary hypotheses were fully supported through converging quantitative and qualitative evidence. Experimental group participants demonstrated significantly steeper developmental growth trajectories across all executive function components compared to both active and passive control groups over the 18-month study period, indicating enhanced rate of skill acquisition in response to the intervention. Working memory capacity improvements averaged 1.1 additional items compared to control groups (experimental group gain: 2.2 items from baseline of 3.2 to final 5.4; passive control gain: 1.2 items from 3.1 to 4.3). Inhibitory control accuracy increased by 6.9 percentage points more in the experimental group (13.7% gain from 72.4 to 86.1%) compared to passive control (7.4% gain from 71.8 to 79.2%). Cognitive flexibility scores showed 1.8-point advantages (experimental gain: 3.4 points from 6.8 to 10.2; passive control gain: 1.5 points from 6.9 to 8.4) that persisted through the 6-month follow-up period.

The neuroplasticity hypothesis received robust support through ERP evidence showing enhanced neural efficiency in the experimental group. These neural changes remained stable post-intervention, indicating that the intervention established enduring alterations in brain organization that continue supporting cognitive development beyond active treatment periods. The mediating role of engagement and attention regulation was confirmed through qualitative findings showing 78% high enthusiasm ratings in experimental group compared to 31% in passive control, with behavioral observations documenting increased sustained attention and reduced impulsivity. The moderating effect of baseline executive function abilities was supported, with children demonstrating initially lower executive function scores showing greater absolute gains (*β*_3_ = −0.34, *p* = 0.012), confirming that the intervention was particularly beneficial for children with initial difficulties.

### 4.2 Theoretical contributions and practical significance

This research makes substantial theoretical contributions to understanding the intersection of narrative engagement, gamification, and cognitive development during sensitive developmental periods. Building upon the foundational work of Posner, Rothbart, Diamond, Dehaene, Jaeggi, and others who have established the importance of experience-dependent plasticity and cognitive training paradigms, this study extends existing neuroplasticity frameworks by demonstrating how narrative-based gamification can serve as an ecologically valid vehicle for promoting executive function development. The integration of neuroplasticity theory with interactive literary approaches provides an applied framework for understanding how experience-dependent brain changes can be optimized through carefully designed educational interventions that leverage children’s natural engagement with stories. The demonstration that narrative-based gamification produces measurable neural modifications advances our understanding of the biological mechanisms underlying cognitive development and learning.

The practical implications are significant for early childhood education policy and practice. The sustained effectiveness observed across the 18-month study period suggests that interactive literary narrative gamification appears to be an effective approach for promoting executive function development through gamified narrative strategies in educational settings. While our observational data documented improved executive function-related behaviors in classroom contexts, we note that claims of far transfer to untrained cognitive domains remain debated in the literature. However, as no direct comparisons with other established executive function intervention protocols were conducted in this study, we cannot conclude that this approach is superior to alternative interventions such as structured cognitive training programs or other game-based approaches. Future research should include comparative effectiveness studies examining multiple intervention types to determine relative efficacy and identify optimal characteristics for different populations and contexts. The accessibility and scalability of technology-based interventions make this approach particularly valuable for addressing individual differences and providing personalized learning experiences that adapt to children’s developmental needs, though implementation requires consideration of resource availability and teacher training requirements (Clark and Mayer, 2016).

### 4.3 Research limitations and future directions

Several limitations constrain the generalizability and interpretation of these findings. The sample was primarily drawn from middle-income families in urban settings, limiting generalizability to diverse socioeconomic and cultural contexts. Future research should examine intervention effectiveness across broader demographic ranges and cultural backgrounds to establish universal applicability. Additionally, the 18-month follow-up period, while substantial, may not capture the full extent of long-term developmental impacts that could emerge over multiple years.

Recent studies have demonstrated the effectiveness of gamified interventions for enhancing literacy and cognitive skills in educational settings (Pasqualotto and Venuti, 2020; Anderle et al., 2025; Cattoni et al., 2024), supporting the potential of technology-based approaches for addressing diverse learning needs.

The reliance on ERP measures as the primary neuroplasticity indicator represents another limitation, as these methods provide limited spatial resolution for understanding specific brain region changes. Future investigations incorporating structural and functional magnetic resonance imaging could provide more comprehensive understanding of neural mechanisms and identify optimal intervention targets within developing brain networks.

### 4.4 Significance for education and neuroscience

This investigation establishes interactive literary narrative gamification as an evidence-based approach for promoting cognitive development during sensitive developmental periods. The convergence of behavioral and neural evidence provides unprecedented insight into the mechanisms through which educational interventions can optimize brain development and learning outcomes. The demonstration that relatively brief interventions can produce lasting neural and cognitive changes has profound implications for early intervention strategies and educational resource allocation (Heckman, 2006).

For the broader neuroscience community, this research advances understanding of experience-dependent plasticity during human development and demonstrates the potential for educational interventions to serve as tools for studying brain-behavior relationships. The integration of longitudinal, experimental, and neurobiological approaches represents a methodological advance that can inform future investigations of learning and development across multiple domains and age ranges.

### 4.5 Educational implications and implementation

The sustained effectiveness of interactive literary narrative gamification suggests significant potential for early childhood education practice. Implementation could occur in preschool classrooms through trained teachers using structured protocols, with optimal frequency of 30-min sessions five times per week based on our findings.

Professional development programs should focus on narrative engagement techniques, adaptive difficulty adjustment, and progress monitoring strategies. The technology platform’s scalability enables individualized learning experiences while maintaining classroom integration. Cost-effectiveness analyses indicate favorable ratios compared to traditional executive function interventions, particularly given the sustained benefits observed through follow-up periods.

Future implementation should consider inclusive settings serving children with developmental differences, as gamified interventions may provide particular benefits for executive function challenges in special educational needs populations.

## Data Availability

The original contributions presented in the study are included in the article/Supplementary material, further inquiries can be directed to the corresponding author.
