# Supplementary material for: The impact of interactive literary narrative gamification on executive function development in preschool children: a longitudinal mixed-methods study based on neuroplasticity theory
Source: Front Psychol. 2026 May 8;17:1656625. doi: 10.3389/fpsyg.2026.1656625 (PMC13193897; doi:10.3389/fpsyg.2026.1656625)
Supplement: Supplementary file 1 [file Table_1.DOCX]

# Supplementary Materials

## Supplementary Table S1. Complete Longitudinal Executive Function Outcomes with Detailed Statistics

| Time Point | Measure | Experimental (n=60) | Active Control (n=60) | Passive Control (n=60) | F-value | p-value | Post-hoc Comparisons | Effect Size (η²) |
| --- | --- | --- | --- | --- | --- | --- | --- | --- |
| **Baseline** | Working Memory Span | 3.2 ± 0.8 | 3.4 ± 0.9 | 3.1 ± 0.7 | 1.89 | 0.154 | n.s. | 0.02 |
|  | Inhibitory Control (%) | 72.4 ± 12.8 | 74.1 ± 11.9 | 71.8 ± 13.2 | 0.68 | 0.509 | n.s. | 0.01 |
|  | Cognitive Flexibility | 6.8 ± 2.1 | 7.1 ± 2.3 | 6.9 ± 2.0 | 0.43 | 0.652 | n.s. | 0.01 |
| **6 months** | Working Memory Span | 4.2 ± 1.1 | 3.8 ± 1.0 | 3.6 ± 1.0 | 5.23 | 0.007 | Exp > PC* | 0.06 |
|  | Inhibitory Control (%) | 78.4 ± 9.2 | 76.1 ± 10.1 | 74.1 ± 10.8 | 2.89 | 0.058 | n.s. | 0.03 |
|  | Cognitive Flexibility | 8.1 ± 2.3 | 7.6 ± 2.2 | 7.2 ± 2.1 | 2.76 | 0.066 | n.s. | 0.03 |
| **12 months** | Working Memory Span | 5.1 ± 1.3 | 4.3 ± 1.2 | 4.0 ± 1.2 | 12.45 | <0.001 | Exp > AC**, PC** | 0.13 |
|  | Inhibitory Control (%) | 84.2 ± 8.6 | 79.8 ± 10.1 | 76.8 ± 11.2 | 8.91 | <0.001 | Exp > AC*, PC** | 0.10 |
|  | Cognitive Flexibility | 9.6 ± 2.0 | 8.5 ± 2.2 | 7.9 ± 2.4 | 9.67 | <0.001 | Exp > AC*, PC** | 0.11 |
| **18 months** | Working Memory Span | 5.4 ± 1.2 | 4.6 ± 1.3 | 4.3 ± 1.4 | 11.28 | <0.001 | Exp > AC*, PC** | 0.13 |
|  | Inhibitory Control (%) | 86.1 ± 7.9 | 82.1 ± 9.5 | 79.2 ± 10.5 | 7.89 | 0.001 | Exp > AC*, PC** | 0.09 |
|  | Cognitive Flexibility | 10.2 ± 1.8 | 9.1 ± 2.1 | 8.4 ± 2.6 | 8.67 | <0.001 | Exp > PC** | 0.10 |

Note: Values are presented as Mean ± SD. Exp = Experimental group; AC = Active Control; PC = Passive Control; n.s. = not significant *p < 0.05; **p < 0.01 (Bonferroni-corrected post-hoc comparisons)

## Supplementary Table S2. Detailed Description of Executive Function Tasks

### Working Memory: Backward Digit Span Task

**Task Description:** Children are presented with sequences of single-digit numbers (1-9) verbally at a rate of one digit per second. They are required to repeat the sequence in reverse order immediately after presentation.

**Administration Procedure:** 1. Practice trials: Two sequences of 2 digits to ensure task comprehension 2. Testing begins with 2-digit sequences and increases by one digit after two successful trials 3. Three trials presented at each span length 4. Task discontinues after failure on all three trials at a given length 5. Total administration time: 5-10 minutes

**Scoring:** Maximum span achieved is recorded as the highest sequence length for which at least two of three trials were correctly recalled. Raw scores range from 2 to 9 digits.

**Materials:** Standardized digit lists, recording sheet, quiet testing environment

### Inhibitory Control: Child-Friendly Flanker Task

**Task Description:** Children view a horizontal array of five fish presented on a tablet screen. The central target fish faces either left or right, while flanking fish (distractors) face either the same direction (congruent trials) or opposite direction (incongruent trials). Children press left or right arrows to indicate the direction of the central fish.

**Administration Procedure:** 1. Practice phase: 12 trials with feedback (6 congruent, 6 incongruent) 2. Testing phase: 60 trials without feedback (40 congruent, 20 incongruent) 3. Stimulus presentation: 500ms with 1500ms response window 4. Inter-trial interval: 1000ms with fixation cross 5. Two blocks of 30 trials with rest period between blocks 6. Total administration time: 10-15 minutes

**Scoring:** Percentage accuracy calculated separately for congruent and incongruent trials. Primary outcome is accuracy on incongruent trials (conflict condition).

**Materials:** Custom software on Samsung Galaxy Tab A tablet, child-sized chair and table

### Cognitive Flexibility: Dimensional Change Card Sort (DCCS)

**Task Description:** Children sort picture cards depicting colored shapes according to one dimension (color or shape), then switch to sorting by the other dimension.

**Administration Procedure:** 1. Pre-switch phase: 6 trials sorting by one dimension (e.g., color) 2. Post-switch phase: 6 trials sorting by opposite dimension (e.g., shape) 3. Cards presented individually on tablet screen 4. Verbal instructions accompany each phase 5. No feedback provided during test trials 6. Total administration time: 8-12 minutes

**Scoring:** Total correct sorts across pre-switch and post-switch phases (0-12 points). Primary outcome is post-switch performance indicating cognitive flexibility.

**Materials:** Tablet-based DCCS software with standardized stimuli (red/blue rabbits/boats)

### Behavioral Regulation: Head-Toes-Knees-Shoulders (HTKS) Task

**Task Description:** Children follow verbal commands requiring them to perform opposite actions (e.g., “touch your head” means touch toes).

**Administration Procedure:** 1. Practice phase: 4 trials with corrective feedback 2. Head-Toes section: 10 test trials (5 “touch head,” 5 “touch toes”) 3. Knees-Shoulders section: 10 test trials (5 “touch knees,” 5 “touch shoulders”) 4. Complex section: 10 trials combining all four commands 5. Each trial scored 0 (incorrect), 1 (self-corrected), or 2 (correct) 6. Total administration time: 10-15 minutes

**Scoring:** Raw scores range from 0-40 based on performance accuracy. Higher scores indicate better behavioral regulation and inhibitory control.

**Materials:** None required beyond verbal instructions and scoring sheet

### Parent Report: BRIEF-P

**Task Description:** Parents complete a 63-item questionnaire rating the frequency of executive function behaviors in daily life contexts.

**Administration Procedure:** 1. Parents receive questionnaire packet with instructions 2. Items rated on 3-point scale: Never (1), Sometimes (2), Often (3) 3. Five subscales: Inhibit, Shift, Emotional Control, Working Memory, Plan/Organize 4. Can be completed at home or during assessment session 5. Completion time: 10-15 minutes

**Scoring:** Raw scores summed for each subscale and total score. T-scores and percentiles calculated using age and gender norms. Higher scores indicate greater executive function difficulties.

**Materials:** BRIEF-P questionnaire, parent instruction sheet, return envelope

## Supplementary Table S3. Semi-Structured Interview Protocols

### Teacher Interview Protocol (Administered at 18-month follow-up)

**Introduction Script:** “Thank you for participating in this research study. This interview will take approximately 30-45 minutes. I’ll be asking you questions about [child’s name]’s behavior and development over the past 18 months. Your responses will be confidential and used only for research purposes. May I audio-record this interview?”

**Section 1: Attention and Focus** 1. How would you describe [child’s name]’s ability to pay attention during classroom activities compared to 18 months ago? - Probes: Can you give me specific examples? In what situations do you notice the biggest changes?

1. Have you noticed any changes in how long [child] can focus on challenging tasks?
   - Probes: What types of activities? How does this compare to peers?
2. Tell me about [child]’s distractibility during learning activities.
   - Probes: What typically distracts them? Has this changed over time?

**Section 2: Self-Control and Impulse Regulation** 4. Describe any changes you’ve observed in [child]’s ability to wait their turn or follow classroom rules. - Probes: Specific situations? Improvements or challenges?

1. How does [child] handle frustration when encountering difficult tasks?
   - Probes: Emotional responses? Problem-solving approaches?
2. Have you noticed changes in impulsive behaviors (e.g., calling out, interrupting)?
   - Probes: Frequency? Contexts where this is most/least evident?

**Section 3: Problem-Solving and Flexibility** 7. Tell me about [child]’s approach to solving problems in the classroom. - Probes: Strategies used? Persistence? Asking for help?

1. How well does [child] adapt when routines change or unexpected situations occur?
   - Probes: Examples? Emotional reactions? Recovery time?
2. Have you observed changes in [child]’s ability to switch between different activities or tasks?
   - Probes: Transition behaviors? Compared to beginning of study?

**Section 4: Social Interactions** 10. Describe any changes in how [child] interacts with peers during group activities. - Probes: Cooperation? Leadership? Conflict resolution?

1. Have you noticed changes in [child]‘s ability to understand others’ perspectives?
   - Probes: Empathy? Sharing? Turn-taking?

**Section 5: Academic Readiness** 12. What changes have you observed in [child]’s readiness for academic tasks (e.g., following multi-step instructions)? - Probes: Specific skills? Compared to classroom peers?

1. How would you describe [child]’s overall progress over the 18-month period?
   - Probes: Biggest changes? Areas of concern? Unexpected developments?

**Closing Questions:** 14. Is there anything else you’d like to share about [child]’s development that we haven’t discussed?

1. Do you have any questions about the study or [child]’s participation?

### Parent Interview Protocol (Administered at 18-month follow-up)

**Introduction Script:** “Thank you for taking time to speak with us today. This interview will take about 30-40 minutes. I’d like to hear about your observations of [child’s name]’s behavior and development at home over the past 18 months. Everything you share will be kept confidential. May I record our conversation?”

**Section 1: Home Routines and Self-Regulation** 1. Tell me about [child]’s ability to manage daily routines (morning, bedtime, meals). - Probes: Changes over 18 months? Specific improvements or challenges?

1. How does [child] handle transitions between activities at home?
   - Probes: Resistance? Tantrums? Cooperation? Compared to before?
2. Describe [child]’s ability to follow instructions or requests at home.
   - Probes: Multi-step directions? Remembering tasks? Need for reminders?

**Section 2: Task Persistence and Problem-Solving** 4. What do you notice about [child]’s ability to stick with challenging tasks (homework, puzzles, building)? - Probes: Frustration tolerance? Asking for help? Giving up?

1. How does [child] approach problems or obstacles at home?
   - Probes: Strategies? Independence? Creativity?
2. Have you observed changes in [child]’s ability to organize their belongings or activities?
   - Probes: Toys, school materials, planning ahead?

**Section 3: Emotional and Behavioral Control** 7. Tell me about [child]’s ability to manage emotions (anger, disappointment, excitement). - Probes: Frequency of outbursts? Recovery time? Self-soothing?

1. How well does [child] wait for things they want (treats, activities, attention)?
   - Probes: Patience? Impulsivity? Changes over time?
2. Describe any changes in [child]’s behavior when playing with siblings or friends.
   - Probes: Sharing? Taking turns? Conflict resolution?

**Section 4: Learning and Memory** 10. What have you noticed about [child]’s memory for information or events? - Probes: Remembering instructions? Recalling stories? Learning new skills?

1. How does [child] engage with stories, books, or educational activities at home?
   - Probes: Attention span? Comprehension? Interest? Changes over time?

**Section 5: Overall Development** 12. What are the biggest changes you’ve seen in [child] over the past 18 months? - Probes: Positive developments? Concerns? Unexpected changes?

1. How would you describe [child]’s readiness for school/next grade level?
   - Probes: Academic skills? Social skills? Independence?
2. Have you noticed any connection between [child]’s participation in the study activities and their development?
   - Probes: Specific behaviors? Interests? Skills?

**Closing Questions:** 15. Is there anything else about [child]’s development you’d like to share?

1. Do you have any questions about the study or the results?

## Supplementary Table S4. Behavioral Coding Schemes for Classroom Observations

### Overview

Systematic time-sampling observations were conducted during 20-minute observation periods using 30-second coding intervals. Two trained observers independently coded behaviors, with inter-rater reliability assessed using Cohen’s kappa.

### Coding Categories and Operational Definitions

**1. On-Task Behavior (κ = 0.88)**

Definition: Child is visually and/or physically oriented toward the assigned activity or teacher instruction.

*Specific Indicators:* - Eyes directed at materials, teacher, or activity - Hands manipulating relevant materials - Body oriented toward work area - Responding appropriately to instructions - Active participation in group discussion

*Non-examples:* - Looking around the room - Playing with non-task materials - Talking about unrelated topics - Physical distance from activity area

*Coding Rule:* Code as on-task if child displays indicators for at least 15 seconds of the 30-second interval.

**2. Enthusiasm/Engagement (κ = 0.82)**

5-point Likert Scale: - **1 (Not Engaged):** No visible interest, withdrawn, resistant to participation - **2 (Minimally Engaged):** Passive compliance, follows directions but shows little interest - **3 (Moderately Engaged):** Active participation with neutral affect - **4 (Engaged):** Active participation with positive indicators (smiling, eagerness) - **5 (Highly Enthusiastic):** Intense positive engagement with multiple indicators

*Specific Indicators for High Enthusiasm (4-5):* - Facial expressions: Smiling, wide eyes, animated features - Verbalizations: Excited comments (“I love this!”), spontaneous questions, eager responses - Body language: Leaning forward, bouncing, hand raising, quick movements toward materials - Sustained attention: Maintains focus without redirection - Initiative: Volunteers, adds ideas, extends activity

*Coding Rule:* Rate overall enthusiasm level for entire 30-second interval based on predominant indicators.

**3. Peer Interaction Quality (κ = 0.85)**

Subcategories: - **Positive Interaction:** Cooperative play, helping, sharing, friendly conversation - **Negative Interaction:** Conflict, grabbing, verbal disagreement, exclusion - **No Interaction:** Solitary play, parallel play without engagement - **Neutral Interaction:** Simple proximity or brief exchanges without clear positive/negative valence

*Specific Indicators - Positive Interaction:* - Sharing materials voluntarily - Taking turns without adult prompting - Helping peer with task - Friendly physical contact (pat, high-five) - Collaborative problem-solving - Initiating friendly conversation

*Specific Indicators - Negative Interaction:* - Grabbing materials from peer - Pushing, hitting, or aggressive contact - Name-calling or teasing - Refusing to share - Excluding peer from activity - Arguing without resolution

*Coding Rule:* Code highest intensity interaction if multiple types occur. Note frequency of each type.

**4. Self-Regulation Behaviors (κ = 0.83)**

Definition: Observable behaviors indicating child’s attempt to manage attention, emotions, or behavior.

*Positive Self-Regulation Indicators:* - Self-talk for task management (“First I do this, then…”) - Deep breathing or visible calming strategies - Seeking appropriate help when frustrated - Self-correction without prompting - Staying in designated area - Following rules without reminders

*Dysregulation Indicators:* - Impulsive actions (blurting, grabbing) - Difficulty remaining seated - Emotional outbursts (crying, shouting) - Throwing materials - Leaving designated area without permission - Requiring multiple redirections

*Coding Rule:* Code presence/absence of regulation and dysregulation separately. Note specific strategies observed.

**5. Narrative-Related Comments (Experimental Group Only) (κ = 0.86)**

Definition: Spontaneous verbal references to story elements, characters, or narrative themes from the intervention.

*Specific Examples:* - Mentioning story characters by name - Referencing plot events from intervention stories - Making connections between current activity and story themes - Using vocabulary from intervention narratives - Discussing problem-solving strategies from stories - Spontaneous storytelling incorporating intervention elements

*Coding Rule:* Tally each distinct narrative reference. Record verbatim when possible.

**6. Adult Interaction (κ = 0.84)**

Subcategories: - **Initiated by Child:** Child approaches teacher/assistant with question, comment, or request - **Initiated by Adult:** Teacher/assistant directs attention to child - **Nature:** Instructional, behavioral management, social/emotional support, routine

*Coding Rule:* Note initiator, nature, and child’s response quality.

### Observation Procedures

**Before Observation:** 1. Review child’s assigned group and identify observation target 2. Position in unobtrusive location with clear view of child 3. Synchronize timing devices between two observers 4. Prepare coding sheets with child ID and date

**During Observation:** 1. Observe for 30-second intervals using silent timer 2. Code immediately following each interval (10 seconds) 3. Repeat for 20-minute observation period (30 intervals total) 4. Maintain objective stance; avoid interaction with children 5. Note environmental factors that may affect behavior

**After Observation:** 1. Complete summary notes within 1 hour 2. Meet with second observer to compare codes 3. Calculate interval-by-interval agreement 4. Resolve discrepancies through discussion and video review if available 5. Submit completed coding sheets to research coordinator

## Supplementary Figure S1. Examples of Activities Implemented Across Study Groups


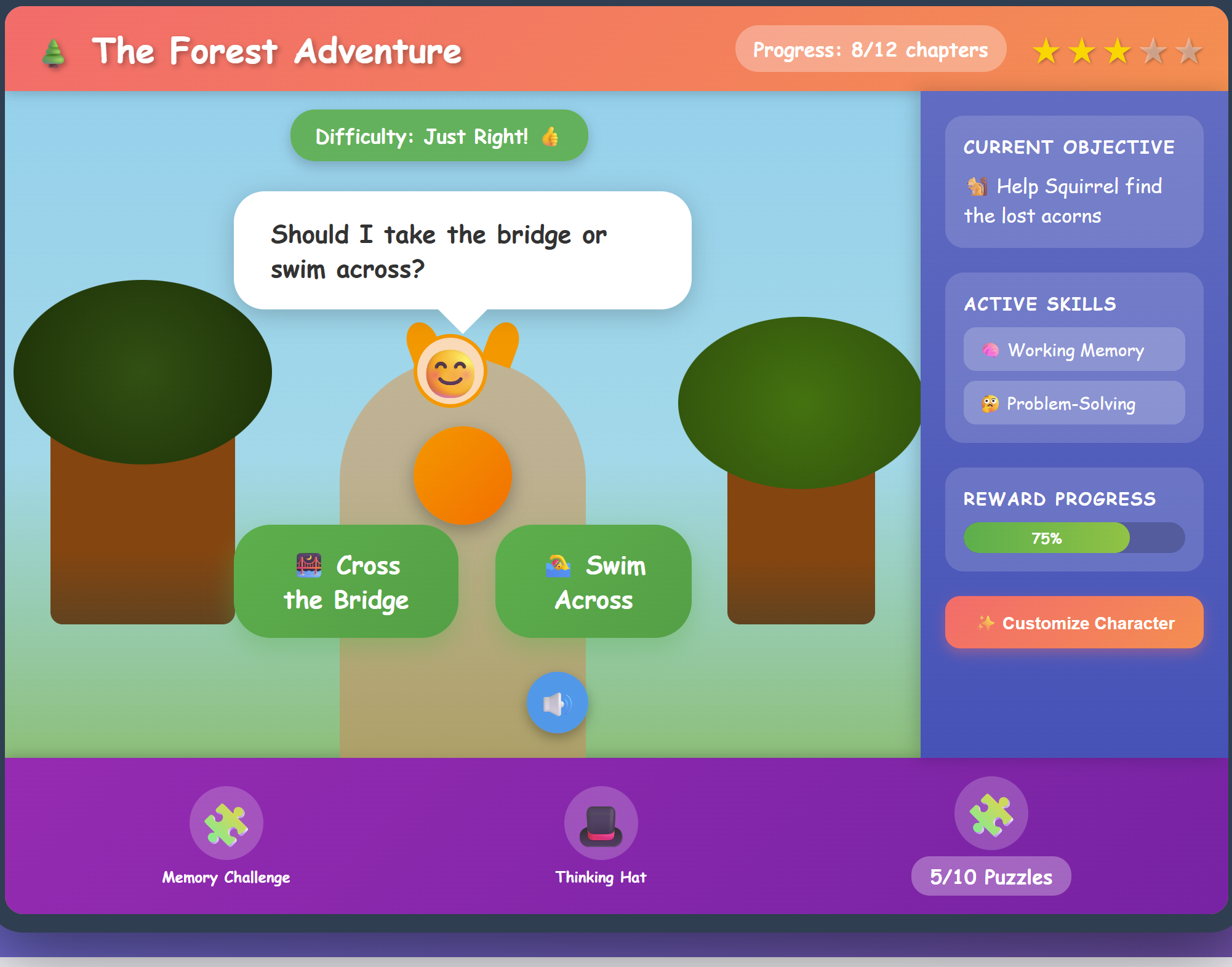


### Panel A: Experimental Group - Interactive Literary Narrative Gamification

###
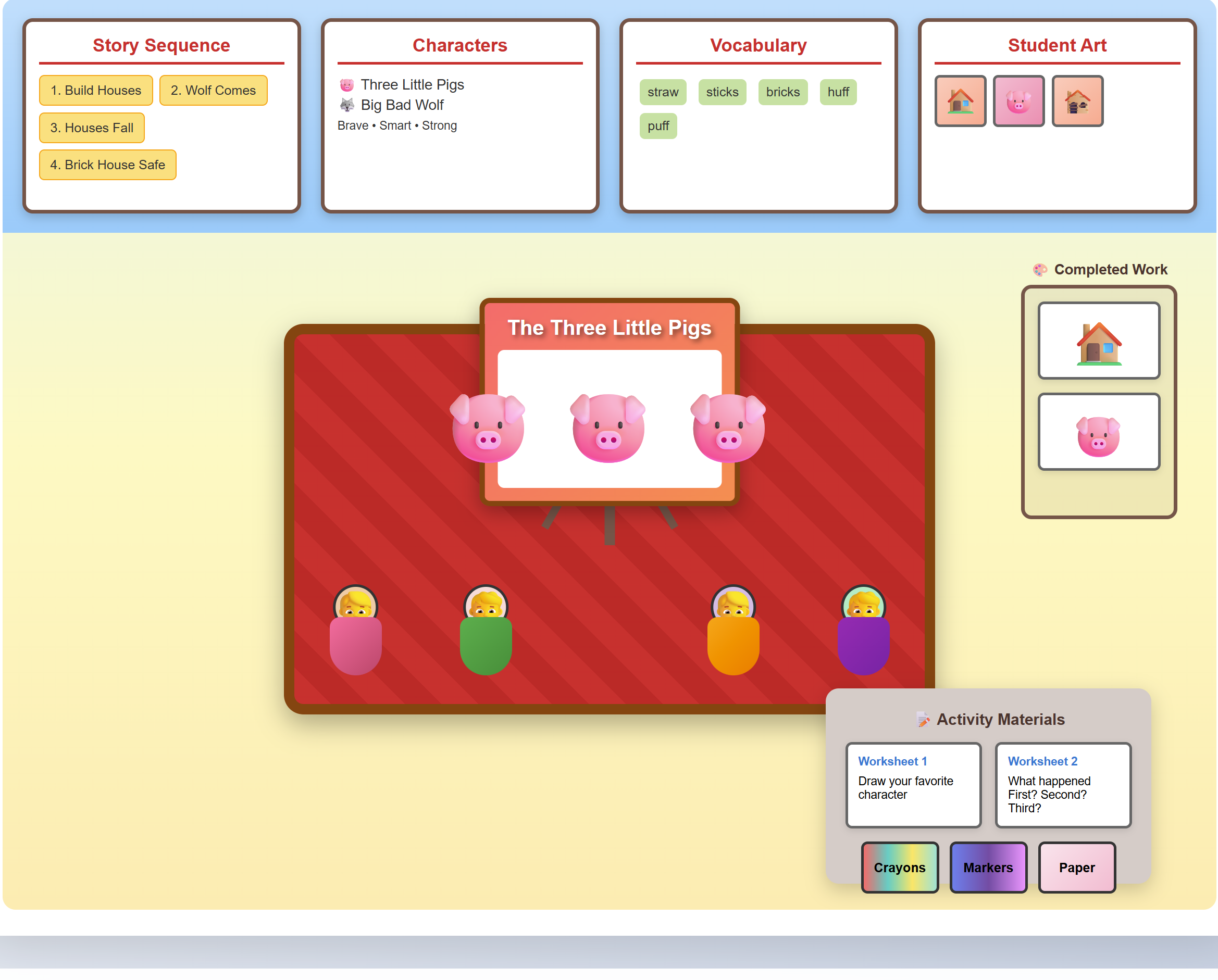


### Panel B: Active Control Group - Traditional Literary Activities

###
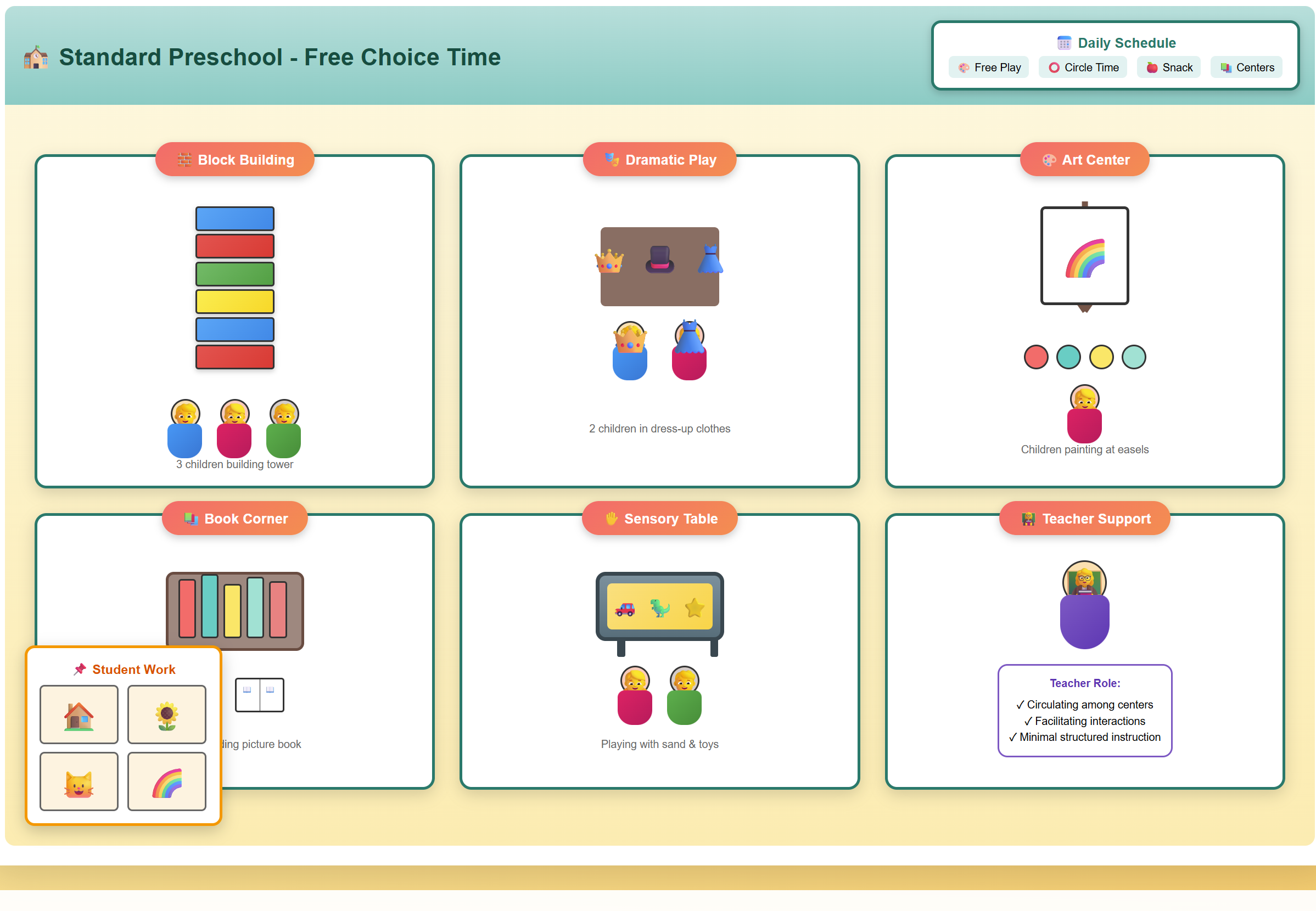


### Panel C: Passive Control Group - Standard Preschool Curriculum

## Supplementary Methods S1. Detailed Scoring Procedures for Each Measure

### 1. Working Memory: Backward Digit Span

**Raw Score Calculation:** - Span level = highest length at which ≥2 of 3 trials correct - If child passes 2/3 trials at length 4 but only 1/3 at length 5, span = 4 - Minimum score: 2 (if no trials passed beyond practice) - Maximum score: 9 (if child successfully completes longest sequences)

**Step-by-step Scoring:** 1. Record child’s verbal response for each trial verbatim 2. Check accuracy: correct order and all digits included 3. Self-corrections within 3 seconds counted as correct 4. Mark trial as pass (1) or fail (0) 5. Calculate number passed at each span level 6. Determine highest span with ≥2 passes 7. Record final span score on summary sheet

**Standardization:** Raw scores used for analysis. Age-normed percentiles available for interpretation but not used in primary analyses.

### 2. Inhibitory Control: Flanker Task

**Raw Score Calculation:** - Separate accuracy calculated for congruent and incongruent trials - Primary outcome = incongruent trial accuracy percentage - Formula: (Correct incongruent trials ÷ 20) × 100

**Step-by-step Scoring:** 1. Software automatically records accuracy and reaction time 2. Export data file after completion 3. Exclude trials with RT < 200ms or > 2000ms (anticipatory or non-responses) 4. Calculate congruent accuracy: (Correct congruent ÷ Total valid congruent) × 100 5. Calculate incongruent accuracy: (Correct incongruent ÷ Total valid incongruent) × 100 6. Calculate interference cost: Congruent accuracy - Incongruent accuracy 7. Record primary outcome (incongruent accuracy) for analyses

**Data Quality Checks:** - Minimum 15 valid incongruent trials required for inclusion - Random responding indicated if accuracy < 30% (exclude case) - Consult with research coordinator if technical issues noted

### 3. Cognitive Flexibility: DCCS

**Raw Score Calculation:** - Pre-switch score: 0-6 points (1 per correct sort) - Post-switch score: 0-6 points (1 per correct sort) - Total score: Sum of pre-switch + post-switch (0-12 maximum)

**Step-by-step Scoring:** 1. Tablet software records each sort as correct/incorrect 2. Review responses for each phase: - Pre-switch (sorting by dimension 1): Count correct sorts - Post-switch (sorting by dimension 2): Count correct sorts 3. Calculate subscores and total score 4. Record qualitative observations: - Perseveration patterns (continuing to sort by pre-switch rule) - Verbal responses indicating understanding - Frustration or confusion behaviors

**Interpretation Guidelines:** - Pre-switch score <4 suggests basic comprehension issues - Post-switch score indicates cognitive flexibility capacity - Perseveration ratio: (Perseverative errors ÷ 6) can be calculated

**Standardization:** Raw total score used for primary analyses.

### 4. Behavioral Regulation: HTKS

**Raw Score Calculation:** - Each trial scored: 0 (incorrect), 1 (self-corrected), 2 (correct) - Self-correction = initial wrong movement followed by correct response within 3 seconds - Maximum score: 40 points (20 trials × 2 points)

**Step-by-step Scoring:** 1. Present verbal command clearly 2. Observe child’s initial response 3. Wait 3 seconds for self-correction before moving to next trial 4. Record score for each trial: - 2 points: Immediate correct response (touches correct body part) - 1 point: Self-corrects within 3 seconds (starts incorrect movement but switches) - 0 points: Incorrect response or no response 5. Sum scores across all 20 trials 6. Calculate subscores if desired: - Head-Toes section (trials 1-10): Max 20 points - Knees-Shoulders section (trials 11-20): Max 20 points

**Special Considerations:** - Partial movements count (e.g., hand moves toward wrong body part but stops) - Verbal self-corrections count (“Oh wait, I mean…”) even without complete movement - Child must touch correct body part for score of 1 or 2

**Standardization:** Raw scores used for analyses. Age-normed percentiles available.

### 5. Parent Report: BRIEF-P

**Raw Score Calculation:** - Sum item responses within each subscale - Calculate composite scores - Convert to T-scores using age and gender norms

**Step-by-step Scoring:** 1. Review completed questionnaire for missing items 2. Contact parent if >2 items missing per subscale 3. Sum items for each clinical scale: - Inhibit (16 items): Items 5, 8, 10, 14, 17, 20, 23, 26, 29, 32, 35, 38, 41, 46, 52, 58 - Shift (10 items): Items 2, 7, 11, 18, 22, 27, 36, 43, 50, 55 - Emotional Control (10 items): Items 1, 4, 9, 15, 21, 28, 33, 40, 45, 53 - Working Memory (17 items): Items 3, 6, 12, 16, 19, 24, 30, 34, 37, 42, 47, 51, 54, 57, 60, 62, 63 - Plan/Organize (10 items): Items 13, 25, 31, 39, 44, 48, 49, 56, 59, 61 4. Calculate index scores: - Inhibitory Self-Control Index (ISCI): Inhibit + Emotional Control - Flexibility Index (FI): Shift + Emotional Control - Emergent Metacognition Index (EMI): Working Memory + Plan/Organize 5. Calculate Global Executive Composite (GEC): Sum of all clinical scales 6. Convert raw scores to T-scores using norm tables (provided in BRIEF-P manual) 7. Interpret T-scores: - T < 60: Within normal limits - T 60-64: Mildly elevated - T 65-69: Potentially clinically elevated - T ≥ 70: Clinically elevated

**Data Quality:** - Inconsistency Index identifies contradictory responses (flag if ≥7) - Negativity Index identifies overly negative response patterns (flag if ≥5) - Review flagged protocols with supervisor

**Standardization:** Both raw scores and T-scores recorded. Raw scores used for primary analyses to maximize sensitivity to change.

### 6. ERP Components: N2 and P3

**Preprocessing Steps:** 1. Import raw EEG data into MATLAB EEGLAB toolbox 2. Apply 0.1-30 Hz bandpass filter (Butterworth, 4th order) 3. Re-reference to averaged mastoids 4. Segment data into epochs (-200ms to 800ms relative to stimulus onset) 5. Baseline correction using -200 to 0ms pre-stimulus interval 6. Automatic artifact rejection: - Voltage threshold: ±100μV - Probability threshold: 5 SD - Kurtosis threshold: 5 SD 7. Manual review of remaining epochs for blinks, eye movements, muscle artifacts 8. Reject epochs with artifacts 9. Calculate minimum 60 artifact-free trials per condition required

**N2 Component Scoring:** - Time window: 250-400ms post-stimulus - Electrode sites: Fz, FCz (frontal) - Measurement: Peak negative amplitude within window - Calculation: 1. Average waveforms across trials for each electrode 2. Identify most negative peak in 250-400ms window 3. Record amplitude (μV) and latency (ms) 4. Average across Fz and FCz for final N2 amplitude score - Expected values: -3 to -7 μV (more negative = stronger response)

**P3 Component Scoring:** - Time window: 300-600ms post-stimulus - Electrode sites: Pz, CPz (parietal) - Measurement: Peak positive amplitude within window - Calculation: 1. Average waveforms across trials for each electrode 2. Identify most positive peak in 300-600ms window 3. Record amplitude (μV) and latency (ms) 4. Average across Pz and CPz for final P3 amplitude score 5. Latency = time point of peak amplitude - Expected values: 4-8 μV amplitude, 350-450ms latency

**Theta Band Coherence Scoring:** - Frequency band: 4-7 Hz - Electrode pairs: Frontal (Fz, FCz) to Parietal (Pz, CPz) - Time window: 0-800ms post-stimulus - Calculation: 1. Compute power spectral density using Welch’s method 2. Calculate coherence between frontal-parietal pairs 3. Average coherence values in 4-7 Hz band 4. Average across time window - Values range 0-1 (higher = stronger connectivity)

**Quality Control:** - Signal-to-noise ratio must exceed 3:1 - Visual inspection of grand average waveforms - Component peaks must be identifiable - Exclude participants with <60 artifact-free trials

**Standardization:** Raw amplitude and latency values used for analyses. Individual differences in baseline values accounted for through statistical modeling.

### General Notes on Scoring Procedures

**Missing Data Handling:** - Single missing item in multi-item scales: Use mean of completed items if <20% missing - Entire measure missing: Use multiple imputation if MCAR assumption met - Document all missing data and reasons

**Inter-rater Reliability:** - 20% of assessments double-scored - Maintain κ ≥ 0.80 for all direct observation measures - Monthly calibration meetings for research assistants

**Data Entry:** - Double-entry verification for all quantitative scores - Range checks programmed into database - Weekly quality control audits

**Scoring Queries:** - Unclear responses reviewed by senior research assistant - Ambiguous cases discussed in weekly team meeting - Documentation of all scoring decisions maintained
